# The solution configurations of inactive and activated DntR have implications for the sliding dimer mechanism of LysR transcription factors

**DOI:** 10.1038/srep19988

**Published:** 2016-01-28

**Authors:** Michael Lerche, Cyril Dian, Adam Round, Rosa Lönneborg, Peter Brzezinski, Gordon A. Leonard

**Affiliations:** 1 Structural Bioloy Group, European Synchrotron Radiation Facility (ESRF), CS 40220, 38043 Grenoble Cedex 9, France; 2 Institut de Biologie Structurale Jean-Pierre Ebel, 71 avenue des Martyrs, CS 10090, 38044 Grenoble Cedex 9, France; 3 European Molecular Biology Laboratory, Grenoble Outstation, 38042 Grenoble Cedex 9, France; 4 Unit for Virus Host-Cell Interactions, University Grenoble Alpes-EMBL-CNRS, 38042 Grenoble Cedex 9, France; 5 Department of Biochemistry and Biophysics, Arrhenius Laboratories for Natural Sciences, Stockholm University, SE-106 91 Stockholm, Sweden

## Abstract

LysR Type Transcriptional Regulators (LTTRs) regulate basic metabolic pathways or virulence gene expression in prokaryotes. Evidence suggests that the activation of LTTRs involves a conformational change from an inactive compact *apo-* configuration that represses transcription to an active, expanded *holo*- form that promotes it. However, no LTTR has yet been observed to adopt both configurations. Here, we report the results of structural studies of various forms of the LTTR DntR. Crystal structures of *apo*-DntR and of a partially autoinducing mutant H169T-DntR suggest that active and inactive DntR maintain a compact homotetrameric configuration. However, Small Angle X-ray Scattering (SAXS) studies on solutions of *apo*-, H169T- and inducer-bound *holo*-DntR indicate a different behaviour, suggesting that while *apo*-DntR maintains a compact configuration in solution both H169T- and *holo*-DntR adopt an expanded conformation. Models of the SAXS-obtained solution conformations of *apo*- and *holo*-DntR homotetramers in complex with promoter-operator region DNA are consistent with previous observations of a shifting of LTTR DNA binding sites upon activation and a consequent relaxation in the bend of the promoter-operator region DNA. Our results thus provide clear evidence at the molecular level which strongly supports the ‘sliding dimer’ hypothesis concerning LTTR activation mechanisms.

LysR Type Transcriptional Regulators (LTTRs), the largest family of transcription factors found in prokaryotes^1^,^2^, are involved in the regulation of basic metabolic pathways or virulence gene expression^3^ and share a high degree of structural identity^4^. LTTR monomers (Fig. 1a) comprise approximately 300 amino acid residues containing two principle domains connected by a linker helix: a large C-terminal Regulatory Domain (RD) and a smaller N-terminal winged Helix Turn Helix (wHTH) DNA Binding Domain (DBD). LTTR RDs comprise two Rossmann fold-like sub domains (RD1, RD2). Ligand binding by inducer molecules which activate LTTRs occurs in Inducer Binding Cavities (IBCs) primarily located at the RD1-RD2 interface^5^,^6^,^7^.

LTTRs generally associate as homotetramers^4^,^8^ in which two DBD dimers flank a central RD tetrameric core (Fig. 1) and regulate the expression of operon genes by binding a divergent promoter region on three different functional subsites: a high affinity Repression Binding Site (RBS), often found near position −65 relative to the transcription start site and two low affinity Activation Binding Sites (ABS’ and ABS”) found near positions −10 and −35 respectively^9^,^10^. Interaction with all three binding sites is essential for the regulation of gene expression by homotetrameric LTTRs. In the current hypothesis – the so-called ‘sliding dimer’ mechanism – LTTR activation leads to a shift in promoter region binding sites from RBS/ABS’ to RBS/ABS”, releasing the −35 box of the promoter region DNA for RNA polymerase recognition and subsequent gene expression.

While there is much biochemical, biophysical and modelling evidence^9^,^10^,^11^,^12^,^13^ to support the sliding dimer mechanism, definitive evidence at the molecular level is scarce. The current hypothesis^6^,^7^ is that upon activation LTTRs morph from a compact to an expanded homotetrameric configuration (Fig. 1b–d)) thus allowing the DBD dimers to bind to different DNA sites. However, while the crystal structures (Fig. 1b,c) of various full-length LTTR homotetramers adopt either compact (*i.e.* CbnR^14^, DntR^5^, BenM^15^) or expanded configurations (*i.e.* TsaR^7^, ArgP^12^, AphB^16^, OxyR^13^) no full-length LTTR homotetramer has yet been observed to adopt both. Moreover, in the only case where the crystal structures of a LTTR are available in both *apo*- and inducer-bound forms (TsaR^7^) both exhibit the same expanded configuration.

To provide further insights into the sliding dimer hypothesis and the conformational changes this might involve, we carried out structural studies of the LTTR DntR, obtaining the crystal structures of inactive *apo-*DntR and of a partially autoinducing H169T mutant (H169T-DntR). Furthermore, we determined, using Small Angle X-ray Scattering (SAXS), models of the solution states of *apo*-, H169T- and inducer-bound (i.e. fully activated, salicylate-bound) *holo*-DntR homotetramers. The crystal structures of *apo*- and H169T-DntR suggest that both inactive and activated DntR homotetramers have the same compact configuration and that no large conformational changes are required for DntR activation. However, SAXS studies of *apo*-, H169T- and *holo*-DntR reveal a completely different picture, suggesting that in solution *apo*-DntR maintains a compact quaternary configuration but that both H169T-DntR and *holo*-DntR homotetramers adopt an expanded conformation. Moreover, putative models bound to promoter DNA regions appear to support the physiological relevance of the solution conformations of *apo*- and *holo*-DntR obtained. These models suggest that a shift of binding sites, accompanied by a relaxation of DNA bend, would occur upon activation of DntR. Our results show that a single LTTR can adopt different conformations, depending on activation state, providing compelling structural evidence to support the sliding dimer mechanism for the activation of homotetrameric LTTRs and confirming that this involves a change in quaternary structure from compact to expanded conformations.

## Results and Discussion

### The crystal structures of *apo*-DntR and H169T-DntR

The crystal structure of *apo*-DntR is isostructural with those of DntR in complex with either acetate or thiocyanate (Table 1, Fig. 2)^5^. As for acetate- or thiocyanate-bound DntR, the asymmetric unit (a.u) contains two DntR molecules with a homotetramer adopting a compact configuration (Fig. 2a) being constructed by the association of two symmetry-related dimers. However, in contrast to the crystal structures of acetate/thiocyanate-bound DntR the crystal structure reported here shows well-defined electron density for the wHTH regions. The crystal structure of *apo*-DntR thus represents the first of a full length DntR homotetramer, although this is extremely similar to the model of a full-length thiocyanate-bound DntR homotetramer^5^ produced by Smirnova and colleagues (r.m.s. deviation in C_α_ positions of 1.04 Å for 1190 residues aligned).

The IBCs in the crystal structure of *apo*-DntR are devoid of any ligands (Fig. 2b), confirming the *apo*- nature of the homotetramers. Indeed, a comparison of the *apo*-DntR IBC conformation with those of the IBCs of ‘open-hinge region’ salicylate-bound DntR RDs^6^ (Fig. 2c) shows that if DntR IBCs maintain the conformation seen in the crystal structure of *apo*-DntR they cannot bind salicylate and DntR cannot be activated. The crystal structure of *apo*-DntR thus seems to confirm that inactive *apo-*DntR homotetramers adopt a compact homotetrameric configuration. Interestingly, a comparison of the configuration of *apo*-IBCs with those of the IBCs seen in the crystal structures of acetate- or thiocyanate-bound DntR (Fig. 2d) shows them to be very similar. This suggests that acetate/thiocyanate molecules bound in IBCs of DntR homotetramers serve to reinforce the *apo-* IBC configuration rather than, as previously suggested^5^, acting as mimics of bound salicylate.

The crystals of H169T-DntR we obtained show very similar unit cell dimensions and the same space group to those of *apo*-DntR (Table 1). Similar to *apo*-DntR, in the crystal structure of H169T-DntR contains a dimer in the a.u. with a homotetramer adopting a compact configuration formed by the association of two symmetry-related dimers. The quality of the electron density map for H169T-DntR did not allow the building of the wHTH regions, hence the final model obtained essentially includes only that of a thiocyanate-bound (Fig. 2e) H169T-DntR RD homotetramer. This adopts an identical quaternary structure to the central RD homotetramer seen in crystals of *apo*-DntR (r.m.s.d. in C_α_ positions, 0.36 Å; 428 atoms superposed in the RD dimers seen in the a.u.). However, a superposition of the crystal structure of H169T-DntR with that of ‘open-hinge’ inducer-bound DntR RDs^6^ (Fig. 2f) shows, in contrast to what we report for *apo*-DntR above, the conformations of their IBCs to be very similar. This observation seems to provide a rational explanation as to why the H169T mutation autoinduces transcription (Fig. 2g) and suggests that upon activation DntR homotetramers could maintain a compact configuration.

### The solution conformations of *apo*- and H169T-DntR

The conclusion, based on the crystal structures described above, that both inactive and active forms of DntR homotetramers maintain a compact configuration is inconsistent with the results of thermal stability analyses (TSAs) of *apo-*, H169T- and *holo-*DntR (Table 2). These show the melting temperature (T_m_) of H169T-DntR is increased by 9 °C relative to that of *apo*-DntR, suggesting that, in solution, there are larger conformational differences between *apo*- and H169T-DntR homotetramers than are seen in the crystal structures described above.

It is generally accepted that, in many cases, the packing interactions required for the formation of crystals can lock a particular macromolecule in a single conformation and there are an increasing number of examples where solution structures reveal physiologically relevant conformations of biological macromolecules that are unobtainable in crystals^17^,^18^,^19^,^20^. We therefore examined the crystal packing interactions for both *apo-* (Fig. 3a) and H169T-DntR and found these interactions constrain the H169T-DntR tetramer to adopt a compact configuration similar to that adopted by *apo-*DntR. In order to verify whether these compact configurations represent the physiological states of *apo*-DntR and H169T-DntR homotetramers, we carried out SAXS experiments (Table 2, Figs 3,4 and 5).

**Figure 1:**
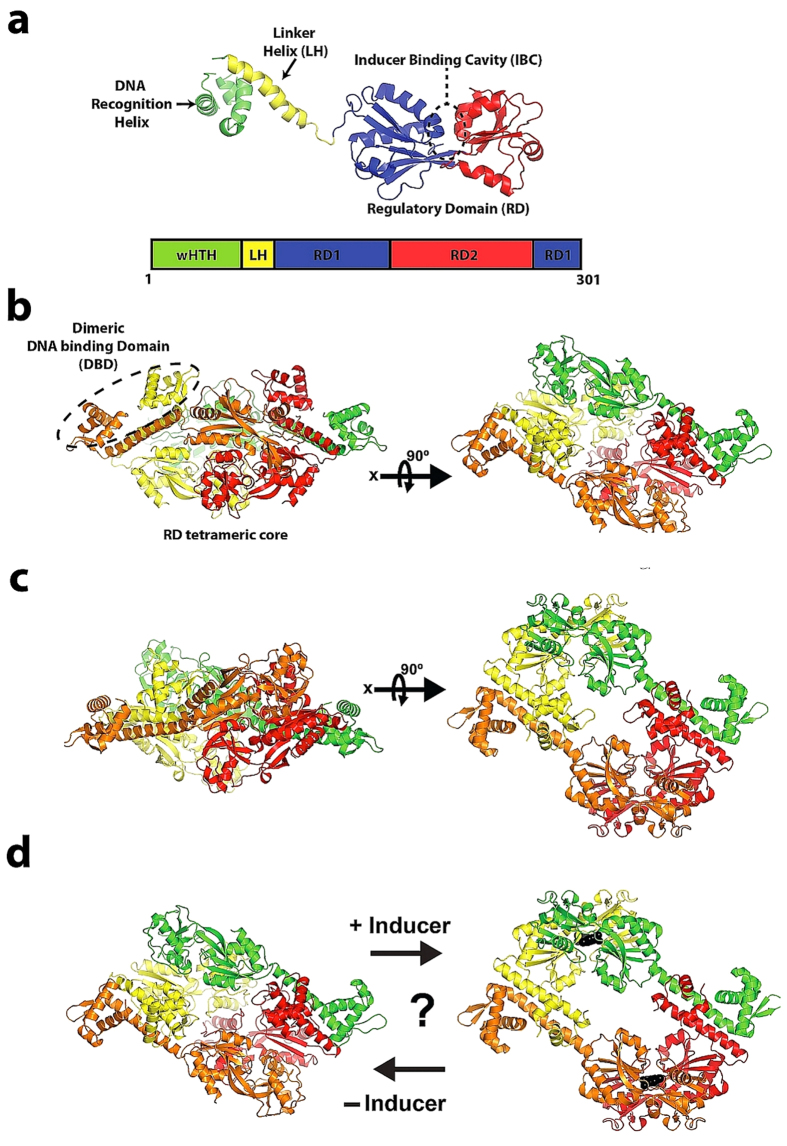
Orthogonal views of the compact and extended tetrameric configurations seen in the crystal structures of full length LTTRs. (**a**) The domain structure of LTTR monomers. Major elements are labelled. (**b**) The crystal structure of CbnR^14^ showing LTTR homotetramers (each monomer coloured differently) in a compact configuration. The position of one of the wHTH dimers that flank the central tetrameric RD core is highlighted. (**c**) The crystal structure of TsaR^7^ showing LTTR homotetramers in an extended configuration. (**d**) The current hypothesis is that activation of LTTRs leads to a change in conformation from compact to expanded homotetrameric configurations.

For *apo*-DntR homotetramers in solution, the Guinier region of the scattering curve obtained yields a radius of gyration of 39 Å and analysis of the pair distribution function (P(r)) derived from the scattering curve obtained suggests a maximum intramolecular distance of 118.2 Å. This latter is significantly shorter than the maximum distance (130.5 Å) observed in the *apo-*DntR crystal structure and suggests, as does the poor agreement (χ^2^ = 2.1) of a fit (Fig. 4a) of the experimentally measured scattering curve and a scattering curve calculated using the homotetramer seen in the crystal structure, that the conformation of *apo*-DntR homotetramers in solution is different to that observed in the crystal. To test whether, in solution, *apo*-DntR homotetramers adopt an expanded configuration form similar to that seen in the crystal structure of TsaR^7^ (Fig. 1b) we constructed a homology model of such a homotetramer using Swiss-Model^21^. A fit of the calculated scattering curve based on this model and the experimental scattering curve also results in a rather poor agreement (χ^2^ = 1.9, Fig. 4a). Therefore, in order to obtain a model of the conformation of *apo*-DntR homotetramers in solution we employed rigid body refinement procedures. The resulting model provides very good fits to both the *ab-initio* molecular envelope obtained from our SAXS experiments (Fig. 5a) and to the experimental scattering curve (χ^2^ = 1.0; Fig. 4a) and shows that in solution *apo*-DntR maintains a compact tetrameric conformation. However, and as expected from the value of D_max_ derived from the P(r) function, the solution shape of *apo*-DntR is less elongated than is seen in the crystal with the DBD dimers – the positions of which may also be influenced by lattice constraints in the crystal - packed closer to the tetrameric core (Fig. 5a,b).

In SAXS analysis of solutions of H169T-DntR the Guinier region of the scattering curve obtained yields a radius of gyration value of 42 Å, larger than the 39 Å observed in solution for *apo*-DntR. D_max_ (135 Å) as derived from the pair distribution (P(r)) function is also significantly larger than that seen in solution for *apo*-DntR (118 Å). SAXS experiments thus indicate that the solution shape of H169T-DntR homotetramers is different to that of inactive *apo-*DntR homotetramers. This observation is reinforced by the results of fits of the experimental scattering curve of H169T-DntR to curves calculated from various structural models (Fig. 4b). Here, calculated curves based on either the solution (χ^2^ = 3.4) or crystal (χ^2^ = 2.4) structures of *apo*-DntR reported here result in very poor matches. However, a scattering curve based on our homology model of a TsaR-type expanded *apo*-DntR homotetramer provides an improved fit (χ^2^ = 1.5). This suggests that in solution H169T-DntR adopts a conformation similar to that of the expanded homotetramers seen in the crystal structure of TsaR. As H169T-DntR activates transcription even in the absence of its inducer molecule salicylate, implying that it more easily adopts an activated conformation than does *apo*-DntR, these results indicate that an expanded conformation is the activated state of DntR and, as previously hypothesised^7^, of LTTR family homotetramers in general.

### The solution model of *holo*-DntR

We have been unable to crystallise full-length DntR in the presence of salicylate and therefore examined the solution conformation of *holo*- (i.e. active) DntR in a series of SAXS experiments. The invariant parameters (R_g_, I (0), D_max_, Porod Volume) derived from scattering curves obtained and the resulting P(r) functions are shown in Table 2. Incubation of *apo*-DntR with 20 μM sodium salicylate produces no significant change compared to *apo*-DntR while incubation with either 100 μM or 5000 μM sodium salicylate yields increased values of R_g_ and D_max_. However, values of I(0) and Porod volume in the presence of 5000 μM sodium salicylate increase markedly compared to those obtained for *apo*-DntR, indicating the presence of sufficient inter-particle effects to make this scattering curve unreliable for further analysis. This is in contrast to values obtained following incubation with 100 μM sodium salicylate. The scattering curve (Fig. 3a) obtained for *apo*-DntR incubated with 100 μM sodium salicylate was therefore used in further analysis of the solution conformation of *holo*-DntR.

For *holo*-DntR a fit of the Guinier region (Fig. 3b) of the scattering curve yields a radius of gyration of 41.4 Å, similar to that obtained for H169T-DntR in solution but larger than that seen for *apo*-DntR. D_max_ (142 Å) is also similar to that obtained for H169T-DntR in solution and significantly larger than is seen for *apo*-DntR (Fig. 3c). As for H169T-DntR, comparisons of the experimental scattering curve of *holo*-DntR with scattering curves based on the solution (χ^2^ = 2.2) and crystal structures (χ^2^ = 2.1) of *apo-*DntR homotetramers show poor fits while that with a curve calculated using the TsaR type homology model of a DntR tetramer is clearly improved (χ^2^ = 1.6) (Fig. 4c). This suggests, again as for H169T-DntR, that the solution conformation of *holo*-DntR homotetramers is closer to the expanded configuration observed for the crystal structure of TsaR than to either of the solution or crystal structures of *apo*-DntR described here.

**Figure 2:**
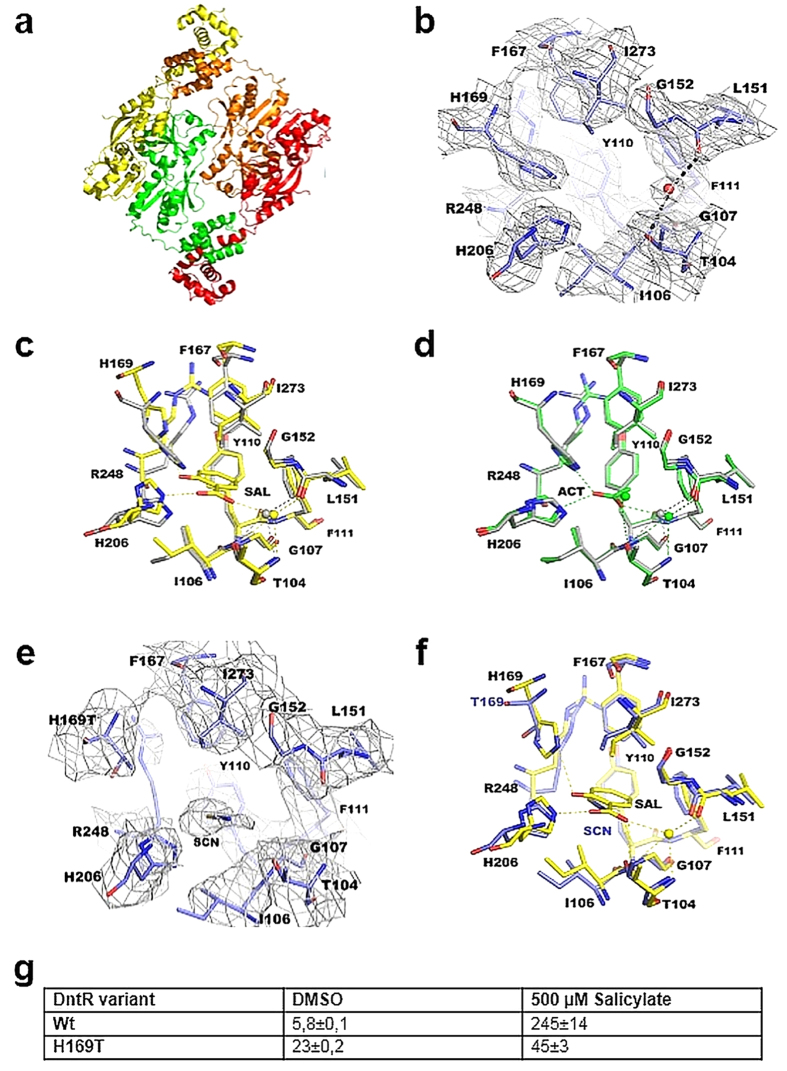
The crystal structures of *apo*- and H169T-DntR. (**a)** The crystal structure of *apo*-DntR with subunits shown in different colours. The homotetramer adopts a configuration with a compact RD core. (**b**) The IBCs in the crystal structure of *apo*-DntR (stick representation) contain only ordered water molecules. Carbon atoms in purple, nitrogen atoms in blue and oxygen atoms in red. Water molecules are shown as red spheres. 2mF_o_-DF_c_, α_calc_ ‘omit’ electron density, contoured at the 1.4 x r.m.s. level, is shown as grey chicken wire. (**c)** Superposition of the *apo*-DntR IBC with that observed in the crystal structure of open-hinge salicylate-bound DntR RDs^6^ (yellow carbon atoms). (**d)** Superposition of the IBCs of *apo*-DntR and acetate-bound DntR^5^ (green carbon atoms). (**e)** The IBCs in the crystal structure of H169T-DntR (stick representation). Carbon atoms in purple, nitrogen atoms in blue and oxygen atoms in red. A thiocyanate ion bound in the IBC shown is labelled. 2mF_o_-DF_c_, α_calc_ ‘omit’ electron density, contoured at the 1.0 x r.m.s. level, is shown as grey chicken wire. (**f)** Superposition of the IBCs in the crystal structures of H169T-DntR and open-hinge salicylate-bound *holo*-DntR RDs^6^. (**g)** The results of flow cytometry measurements show that H169T-DntR autoinduces transcription albeit at a lower level than fully activated wild-type DntR.

**Figure 3:**
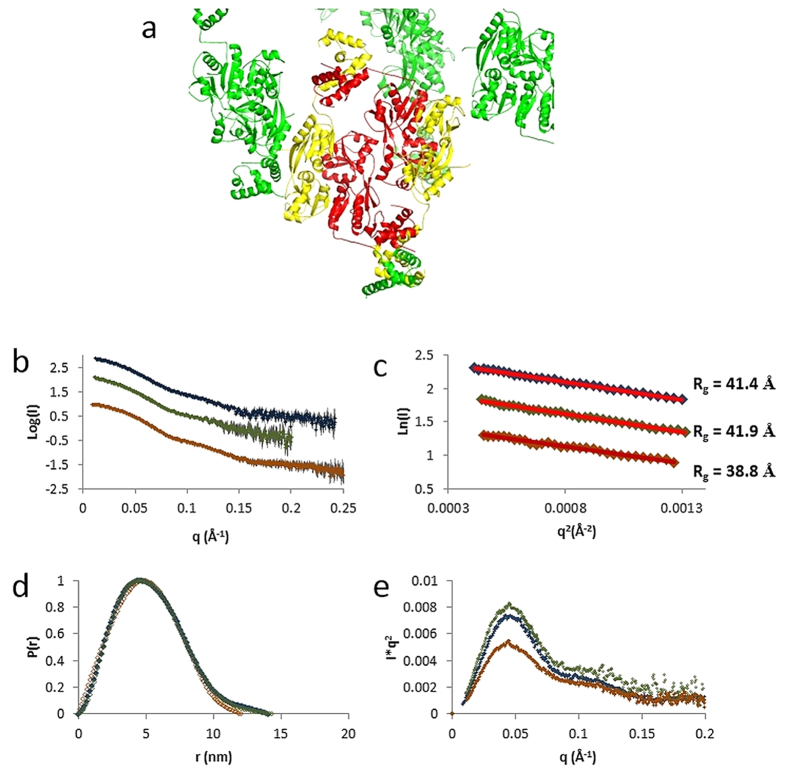
Crystal packing and SAXS analysis of the solution conformations of *apo*-DntR, H169T-DntR and *holo*-DntR. (**a)** An examination of the packing of the crystal form obtained for both *apo-* and H169T-DntR shows that this constrains *apo-* and H169T-DntR to adopt the same compact tetrameric configuration. Here an *apo*-DntR tetramer is shown in red and yellow and symmetry related DntR monomers are shown in green. For clarity not all symmetry-relate monomers in close contact with the tetramer are shown. **(b)** Experimental solution scattering curves (open squares) and the resulting fitted model (solid line) for *apo*-DntR (orange), H169T-DntR (green) and *holo*-DntR (blue). (**c)** Guinier plot regions the fits of which (solid lines, colour scheme as in (**b**)) yield the radii of gyration (R_g_) shown. (**d)** The pair distribution functions P(r) indicating D_max_ for *apo*-DntR (118.2 Å), H169T-DntR (135 Å) *holo*-DntR (142 Å). Colour scheme as in (**b)**. (**e)** Kratky plots (colour scheme as in (**b**)) for *apo*-, H169T- and *holo*-DntR.

**Figure 4:**
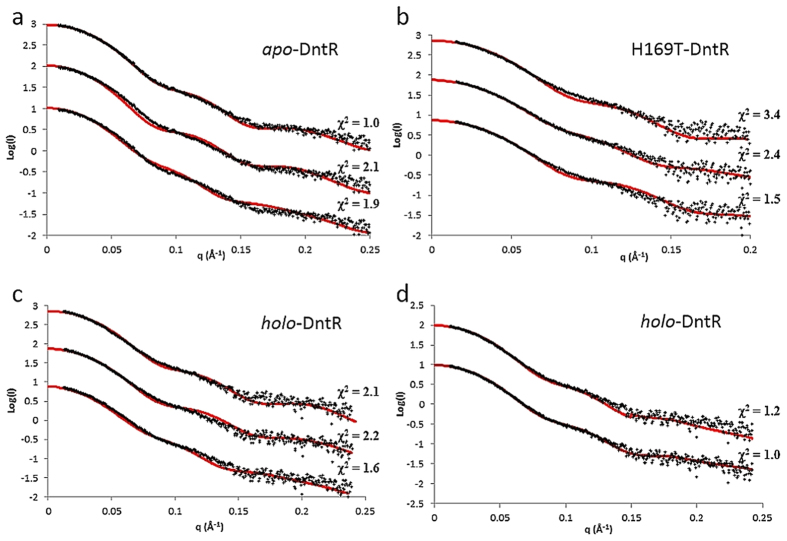
Comparison of the experimental scattering curves of *apo*-, H169T- and *holo*-DntR with theoretical scattering curves based on various structural models. CRYSOL fits of: (**a**) The solution scattering curve of *apo*-DntR with theoretical curves based on (top) the solution conformation of *apo*-DntR shown in Fig. 5a; (middle) the crystal structure of *apo*-DntR shown in Fig. 2a and (bottom) a homology model of an extended, TsaR-type *apo*-DntR homotetramer; (**b**) the solution scattering curve of H169T-DntR with the same theoretical curves shown in (**a**); (**c**) the solution scattering curve of *holo*-DntR with the same theoretical scattering curves shown in (**a**); (**d**) the scattering curve of *holo*-DntR and theoretical curves based on the SASREF-obtained models of solution structures of *holo*-DntR with a central tetrameric core comprising open (top) or closed (bottom) RDs.

**Figure 5:**
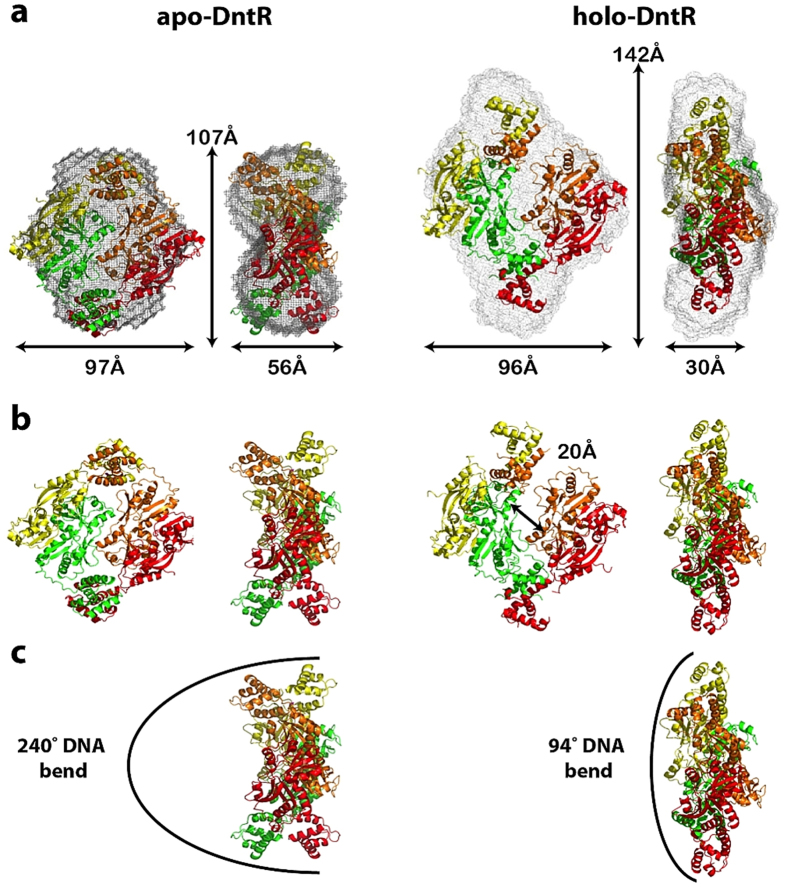
The solution conformations of *apo-* (left) and *holo*-DntR (right). (**a)** Orthogonal views of the *ab initio* molecular envelopes (grey mesh) and the SASREF-obtained models of the solution conformations of *apo*-DntR (P22 symmetry imposed on the molecular envelope) and *holo-*DntR. The dimensions of the SAXS-obtained *ab initio* envelopes are shown. (**b)** Orthogonal views of the SASREF-derived solution conformations of *apo*-DntR and *holo*-DntR. The transition from compact (*apo-*) to expanded (*holo-*) homotetrameric configurations involves an ‘opening’, shown by the black arrow, by ~20Å of the central tetrameric RD core. (**c)** Side views of the SAXS-obtained solution conformations of *apo*- and *holo*-DntR showing the likely bend of bound promoter region DNA.

To obtain a clearer idea of the solution structure of activated *holo-*DntR homotetramers rigid body modelling was employed. While the scattering curve calculated from the resulting model provides a good fit (χ^2^ = 1.2) to the experimental curve (Fig. 4d), poor agreement at higher q values indicates local differences between this model and the true solution structure. A plausible explanation is that the RDs treated as rigid bodies in the modelling procedure derive from the crystal structure of acetate-/thiocyanate bound DntR which, as has been shown above (Fig. 2c,d), cannot bind salicylate. In an attempt to obtain a better fit these were replaced with RDs observed in the crystal structure of doubly-salicylate-bound *holo-*ΔN90DntR (PDB 2Y7K, chain A and B)^6^. Here, the IBCs are expanded to accommodate salicylate and the RD is slightly closed around the ligand. This results in a model for the solution structure of *holo*-DntR which provides an excellent fit to the *ab-initio* molecular envelope obtained (Fig. 5b) and for which the calculated scattering curve provides a very good fit to the experimental scattering curve over the entire q range (χ^2^ = 1.0, Fig. 4d).

Our SAXS experiments thus indicate that, in solution, both H169T-DntR (autoinducing) and *holo*-DntR (fully activated) homotetramers do not adopt the compact conformation seen in solution for *apo*-DntR homotetramers but that both adopt similar, TsaR-type expanded configurations. This observation is consistent with TSA measurements showing that both H169T-DntR and *holo*-DntR have similar T_m_s which are significantly higher than that seen for *apo*-DntR (Table 2). Our SAXS experiments thus show the solution conformation of activated DntR to be that of an expanded form homotetramer with an open central cavity and very reminiscent in nature to the conformation seen in the crystal structure of TsaR.

### Models of the binding of the solution conformations of *apo*- and *holo*-DntR to promoter region DNA

DntR, and other members of the LTTR family, are bound to DNA in both inactive and active states^5^,^11^. Activation of transcription by LTTRs must therefore be coupled to conformational differences between active and inactive states prompted by the binding of inducer molecules. The results of our SAXS studies (Fig. 5) suggest, as previously hypothesised^6^,^7^, that this conformational change involves a transformation from a compact homotetrameric configuration which represses expression to an expanded configuration that promotes it.

Our SAXS studies of *holo*-DntR also suggest, as we have previously postulated^6^, that the driving force for the transition from compact to expanded homotetrameric configurations is a closure upon inducer binding of the RDs making up the core of the tetramer which, if the compact form of the homotetramer were maintained, would result in steric clashes between RD2 subdomains at the tetramer interface. Our SAXS studies also indicate that upon activation, as might be expected from an analysis of the crystal structure of TsaR^7^ which the SAXS-obtained solution conformation of *holo*-DntR closely resembles, DntR homotetramers adopt a much flatter configuration than is seen for *apo*-DntR (Fig. 5). In this activated conformation, the separation between the recognition helices in the HTH dimers flanking the central RD tetrameric core is significantly increased compared to that seen in solution for *apo*-DntR, suggesting both different promoter region DNA binding sites for inactive and activated DntR and a relaxation in the bend of promoter region DNA upon the transition from the inactive to active conformations of DntR.

**Figure 6:**
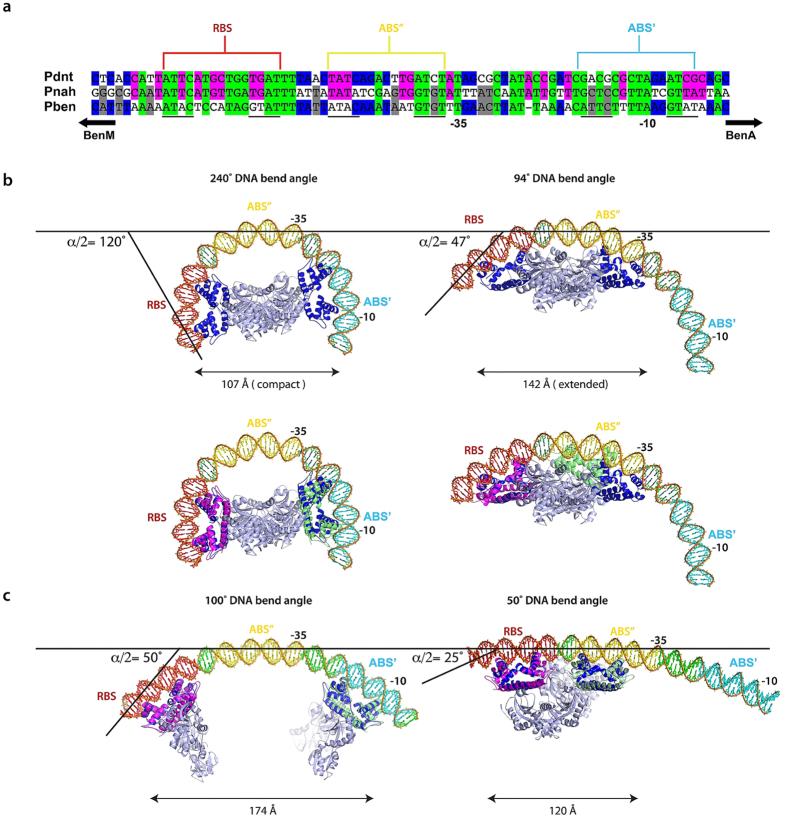
Models of transcriptional activation by homotetrameric LTTRs. (**a)** Alignment of the operator–promoter regions p_dnt_, p_nahr_ and p_benA_, indicates that the DNA binding sites of DntR and BenM are located in similar regions. (**b)** Top: Models of the binding of p_benA_ to the SAXS-obtained solution conformations of inactive (left) and inactive (right) DntR homotetramers indicate that a conformational change from compact (inactive) to more extended (active) homotetramers would result both in a switch in DNA binding sites and a relaxation (240^o^ to 94^o^) in the bend of operator–promoter region DNA. Bottom: As for the top panel but also showing the predicted DNA binding regions for the wHTH domains (magenta and green) of the LTTR BenM. (**c**) Models of p_benA_ with smaller bending angles and how LTTRs might bind to these. Left: For promoter-operator region DNA with a 100^o^ bend, simultaneous binding of RBS and ABS’ (separated by ~174 Å) regions cannot be achieved if a LTTR maintains a homotetrameric conformation. Right: For promoter-operator region DNA with a 50^o^ bend, simultaneous binding of RBS and ABS” (separated by ~120 Å) can be achieved by a homotetrameric LTTR although this will have a conformation very different to that described here for activated (*holo-*) DntR.

In the absence of bound inducer molecules, inactive tetramers of the LTTR BenM binds to two palindromic sites (RBS and ABS’) on p_benA_ promoter region DNA repressing expression. Upon activation, DNA binding sites shift to the RBS and ABS” sites of p_benA_ and transcription occurs^11^. A similar ‘sliding’ of the DNA binding sites of inactive and active LTTRS has also been observed for OccR^22^ and OxyR^23^. While the exact nature of the DntR RBS and ABS’ and ABS” binding sites in its p_dnt_ promoter region DNA have yet to be determined, alignment of p_dnt_ with the nucleotide sequences of p_benA_ and p_nahr_ (the latter the promoter-operator region of another homologous LTTR, NahR) suggests that these will be found in similar regions in all three promoter regions (Fig. 6a). In order to verify that the models obtained here for the solution conformations of *apo*- and *holo-*DntR are physiologically relevant, we constructed models of both species bound to p_benA_.

Here, the 3D-DART server (http://haddock.science.uu.nl/dna/dna.php)^24^ was used to generate series of models of p_benA_ (75bp in length) each with a total DNA bending angle distributed homogenously along the DNA sequence. The most convincing DNA model allowing interaction between the solution structure of *apo*-DntR and both the RBS and ABS’ binding sites of p_ben_A was obtained with a DNA displaying a bend angle of 240° (Fig. 6b). In this model, as seen in the crystal structure of wHTH DNA binding domain (DBDs) of BenM in complex with RBS of p_benA_^25^, the recognition helices and wings of both wHTH dimers interact with consecutive major and minor grooves of each binding site.

A similar approach was performed for *holo*-DntR (Fig. 6b) with the resulting model suggesting that a 94° bending of the p_benA_ region is required to satisfy the simultaneous interaction of its RBS and ABS” binding sites with the two wHTH dimers flanking the heterotetrameric core. Here, however, our model predicts that the ABS” binding region of activated DntR is shifted more towards the −10 region than is predicted for BenM. This observation may explain why no conserved ABS” binding motif has yet been deduced for LTTRs^26^.

The models described above are consistent both with a sliding of DntR promoter region binding sites and a significant relaxation of promoter region DNA bend upon the activation of LTTRs^27^,^28^,^29^ and suggest the solution conformations obtained here for *apo-* and *holo-*DntR are physiologically relevant. However, while the bending angles of bound promoter-operator region DNA (94^o^ for active DntR, 240° for its inactivated counterpart) deduced from our models are not inconsistent with relatively high degrees of DNA bend that can induced in short DNA sequences^30^,^31^,^32^ they are much more pronounced than those measured for LTTRs in circular permutation experiments (0–50° and 50–100° for active and inactivated LTTRs, respectively)^8^. This is intriguing and we therefore constructed a second series of models (Fig. 6c) to try to predict how LTTR homotetramers might bind to DNA promoter-operator regions with lower bending angles. Our model in which the p_benA_ DNA promoter-operator region is bent by 50^o^ (i.e. close to that measured for active LTTR homotetramers) shows that simultaneous binding of RBS and ABS” can be achieved by a homotetrameric LTTR although this will have a conformation very different to those described here for activated or inactivated DntR. Our model of LTTR binding to p_benA_ with a DNA bending angle in the region of 100° (i.e. close to that measured for inactive LTTRs) shows that simultaneous binding of RBS and ABS’ regions cannot be achieved if DntR, and thus other LTTRs, maintain a homotetrameric conformation. Indeed, this model predicts that in their inactive states LTTRs will bind to RBS and ABS’ as dimers and implies that the activation process involves changes in the affinity of a LTTR dimer such that ABS” is preferred for binding over the ABS’ and that binding to the ABS” results in the formation of a LTTR homotetramer. Given that LTTRs generally associate as homotetramers^4^,^8^ when not bound to DNA this scenario appears unlikely and our ‘high-angle’ DNA models (Fig. 6b) thus seem both to confirm the physiological relevance of the solution structures of inactive and activated DntR described here and to indicate, as has been previously suggested^33^, that the bending angles reported for LTTR promoter-operator regions based on circular permutation experiments are underestimated.

Previous studies have shown small basal (i.e. in the absence of inducer molecules) levels of transcription in genes placed upstream of the promoter region controlled by DntR^34^. This might suggest that *in vivo apo*-DntR exists in equilibrium between the compact configurations shown in Fig. 5a and the expanded configuration shown in Fig. 5b. Both our modelling of the DNA binding of the solution conformations we have obtained for the repressor form of DntR (*apo*- state) and activator-form form of DntR (*holo-* form) (see above) and DNA footprinting/binding assays accumulated on LTTRs^11^,^22^,^35^ do not seem consistent with this hypothesis. However, we do not rule out that *apo*-DntR exists in an equilibrium of inactive and active conformations with the fraction of the latter too small to be detected in our SAXS experiments. We also do not rule out that *apo*-DntR can exist in an equilibrium of conformations between a form that completely represses transcription and a form that incompletely represses it. A possibility for a conformation that incompletely represses transcription is that seen in the crystal structures of *apo*-DntR and H169T-DntR reported here. Again, however, we see no evidence for such an equilibrium of conformations in our SAXS analysis of *apo-*DntR.

## Conclusions

The current consensus is that LTTRs regulate transcription through large conformational changes which modify LTTR DNA binding sites promoter region DNA. Previous structural studies^6^,^7^,^12^ have inferred that the change in question is a transition from compact to expanded form tetramers but real structural evidence for this idea has thus far been scarce. In particular, no full-length LTTR homotetramer has yet been observed to adopt both conformations. The results presented here provide the first clear evidence both that a single LTTR homotetramer can adopt both conformations and that the conformation adopted by the homotetramer is a function of activation state. In solution, inactive *apo-*DntR homotetramers adopt a compact configuration in which DBD dimers pack closely against a compact RD tetrameric core (Fig. 5a) while activated DntR homotetramers adopt a more open quaternary configuration (Fig. 5b) highly reminiscent of the configuration seen in the crystal structure of TsaR. That these configurations of active and inactive DntR are physiologically relevant is supported by models of the binding of these two distinct homotetrameric arrangements to promoter region DNA which are consistent both with a sliding of DntR binding sites and a significant relaxation of DNA bend upon activation. Given the level of structural similarity of LTTR family members this work appears to confirm, as previously proposed^12^, that a switch from compact to expanded configurations is likely to be a general activation mechanism for the largest family of transcription factors found in prokaryotes. However, a number of fundamental questions remain to be answered. In particular, the high angles of the bending LTTR promoter region DNA predicted in the models presented here are incommensurate with those measured for other LTTRs in permutation experiments. Further studies will clearly be required to fully validate our conclusions.

## Methods

### Cloning, expression and purification

DntR-His_6_ (DntR) from *Burkholderia sp.* was expressed and purified as previously described^5^ with the exceptions that two pellets of cOmplete Protease Inhibitor Cocktail Tablets (Roche; http://www.roche-applied-science.com) per litre of culture were added during cell lysis and that purification was conducted at 4 ^o^C. To produce H169T-DntR-His_6_ (H169T-DntR) site-directed mutagenesis using PCR primers containing the mutation was carried out (Stratagene, La Jolla, USA; forward primer 5′cggcgcctctttcgcacccgctacgtatgcat3′; reverse primer 5′atgcatacgtagcgggtgcgaaagaggcgccg3′). H169T-DntR was then expressed and purified as for wild-type DntR. Once purified, both proteins were stored at various concentrations in a buffer comprising 1 M NaCl, 2 mM MgSO_4_, 1 mM DTT, 17% (v/v) glycerol, 25 mM NaH_2_PO_4_-NaOH (pH 8.0). These solutions were then used as stocks in both crystallisation protocols and SAXS experiments.

### Crystallisation, data collection, structure solution and refinement

Crystals of *apo*-DntR were grown at 18 ^o^C in hanging-drops by mixing 1 μL of the protein stock solution (~5 mg/mL) with 1 μL of a reservoir solution consisting of 0.2 M sodium potassium tartrate, 0.1 M Tris-HCl pH 8.0, 5% (w/v) PEG 6000. Crystals of H169T-DntR were obtained as previously described for thiocyanate-bound wtDntR^5^ from drops prepared by mixing 1 μL of the protein stock solution with 1 μL of a reservoir solution comprising 0.2 M sodium tartrate, 0.2 M potassium thiocyanate, 0.5 mM sodium salycilate, 0.1 M Tris (pH 8.5) and 20% (w/v) PEG 8000. Crystals were cryoprotected in the relevant reservoir solution supplemented with of 20% (v/v) glycerol and flash frozen in liquid nitrogen. Diffraction data (Table 1) were collected on ID29 (*apo-*DntR) and ID23-1 (H169TDntR) of the European Synchrotron Radiation Facility (ESRF^36^,^37^). Diffraction images were integrated and scaled using XDS^38^, symmetry-related intensities merged using SCALA^39^ and structure factors derived using TRUNCATE^40^. For both *apo*-DntR and H169T-DntR structure solution was carried out using Molecular Replacement (MR) in the program PHASER^41^ using the crystal structure of acetate-bound DntR (PBD ID, 1UTB^5^) stripped of water molecules and other ligands as a search model. Structure refinements (Table 1) were carried out in REFMAC5^42^ and PHENIX^43^ interspersed with rounds of manual rebuilding in COOT^44^. The crystal structure of *apo*-DntR was refined to d_min_ = 2.64 Å resulting in a model providing R_work_ = 19.9% and R_free_ = 22.7%. The crystal structure of H169T-DntR was refined to d_min_ = 3.30 Å and resulted in a model providing R_work_ = 18.8% and R_free_ = 24.0%.

### Small-angle X-ray Scattering (SAXS)

SAXS measurements were carried out on ESRF beamline ID14-3^45^ at λ = 0.931 Å or ESRF beamline BM29^46^ at λ = 0.992 Å. Scattering curves were recorded in the momentum transfer range 0.04 < q < 0.61 Å^−1^ (q = 4π sin (θ)/λ, 2θ is the scattering angle) using a Pilatus 1M detector (Dectris Ltd., Baden, Switzerland). Prior to experiments all solutions were centrifuged at 16,000 xg for 10 minutes to remove aggregated particles. In static SAXS experiments 30 μL of protein solution loaded into a sample capillary using a liquid handling robot were exposed to X-rays and scattering data collected using multiple exposures (see below for details of number and length). Matched buffer measurements taken before and after every sample measurement were averaged and used for background subtraction. Individual exposures were processed automatically and independently using PyFAI^47^ as implemented in the EDNA framework yielding radially averaged curves of normalized intensity versus scattering angle. For each exposure series additional data reduction used the automatic data processing tools of EMBL-Hamburg ATSAS package^48^ to combine individual measurements, exclude any data points affected by aggregation induced by radiation damage, and yield an average scattering curve. Merging of scattering curves obtained at different sample concentrations and downstream analysis were performed manually as described in the literature^49^. Here, the forward scattering I(0) and radius of gyration (Rg) were calculated from the Guinier approximation^50^. Particle volume and the maximum particle size (D_max_) were determined from the pair distribution function P(r) as computed by GNOM^51^ using PRIMUS^52^. Scattering curves from solutions of *apo*-DntR (buffer as for the stock solution described above) were measured at sample concentrations of 3, 1 and 0.5 mg/mL. For all concentrations, images were recorded using 10 exposures each of 10 seconds and the scattering curves obtained at each concentration scaled and averaged to produce the final solution scattering curve. *Ab initio* models of the solution shape of *apo-*DntR were derived from the experimental scattering curve using DAMMIF^53^, imposing P22 symmetry as well as standard P1. To produce a final molecular envelope 20 independently generated *ab initio* models were aligned, averaged and filtered using DAMAVER^54^. Theoretical scattering curves of *apo*-DntR and the TsaR type homology model were calculated and used to generate fits against experimentally-obtained scattering curves using CRYSOL^55^. Rigid body modelling of the model of the solution conformation of *apo-*DntR was carried out in the program SASREF^56^. Here, using the previously postulated model of the structure of a full-length DntR tetramer^5^ as a basis, the four RDs and two DBD dimers that make up a LTTR tetramer (Fig. 1) were treated as separate rigid entities and their positions refined against the experimental scattering curve employing a connectivity restraint of 3Å between residues T85 and A86 of each DntR monomer. Scattering curves from solutions of *apo*-H169T-DntR (same buffer as for the stock solution described above) were measured at a sample concentration of 0.7 mg/mL. 10 × 10 frames each of 2 seconds exposure time were recorded and averaged. Given the limited resolution of the scattering curve obtained rigid body modelling of the solution conformation of *apo*-H169T-DntR was not attempted. The solution shape of *holo*-DntR was elucidated from a scattering curve obtained from a solution of *apo*-DntR pre-incubated with 100 μM sodium salicylate. *Ab initio* models of the solution conformation of *holo*-DntR were obtained as for *apo-*DntR with the exception that no symmetry restrictions were imposed. Rigid body modelling of the solution conformation of *holo*-DntR was carried out as for *apo*-DntR. To verify that results obtained from static SAXS experiments using solutions of *apo-*DntR and of *holo-*DntR were not artefacts due to interparticle interactions, experiments were repeated, this time coupled with size exclusion chromatography (SEC, Viscotek RImax, Malvern; Superdex 200 column) immediately prior to sample injection^57^. Solutions were applied to a gel filtration column and the eluent exposed directly to the X-ray beam. For each solution applied to the column 3500 frames were collected. Individual scattering curves yielding similar values of R_g_ were then processed and averaged. The scattering curves obtained were rather noisy as the concentration of proteins in the eluent is low. Nevertheless, it was possible to obtain satisfactory Guinier regions and P(r) plots from which invariant parameters were derived (Table 2). These are near identical to those obtained in our static SAXS measurements, confirming that models for the solution conformations of *apo-*DntR and of *holo-*DntR derived from higher q-range experiments are valid.

### Thermofluor assays

Thermofluor assays of solutions of *apo*-DntR, *apo*-H169T-DntR and *holo*-DntR were carried out by the EMBL Grenoble Outstation High Throughput Crystallisation Laboratory (https://embl.fr/htxlab/) a platform of the Grenoble Partnership for Structural Biology (PSB; http://www.psb-grenoble.eu/) using a previously published experimental protocol^58^.

### Flow cytometric analysis of whole cell systems

The H169T mutation was introduced to the wild-type DntR gene carried on the plasmid pQEwtdntRHis6:PDNT:gfp using a site-directed mutagenesis kit (Stratagene, La Jolla, CA, USA) according to the manufacturer’s instructions. *E. coli* DH5α cells transformed with the plasmids containing pQEwtdntRHis6:PDNT:gfp or pQEH169TdntR:PDNT:gfp as previously described^34^ were grown overnight in 5 mL LB medium, then inoculated with 1 mL growth media to an OD_600_ of 0.05. After 2 h, 500 μM salicylate in DMSO or only DMSO was added to the cultures. After a further 4 hours cells were taken for Fluorescence-activated Cell Sorting (FACS) analysis performed on a FACS Calibur instrument (BD Biosciences, San Jose, CA, USA). Flow cytometric data were analysed using the FlowJo software, and the mean fluorescence intensity for each cell population was measured.

## Additional Information

**How to cite this article**: Lerche, M. *et al*. The solution configurations of inactive and activated DntR have implications for the sliding dimer mechanism of LysR transcription factors. *Sci. Rep.* **6**, 19988; doi: 10.1038/srep19988 (2016).

## Figures and Tables

**Table 1:** Data processing and structure refinement statistics for *apo*-DntR and H169T-DntR.

	***apo*** **-wtDntR**	**H169T-DntR**
Data collection		
Beamline	ESRF ID29	ESRF ID23-1
Wavelength (Å)	0.976	1.00
Space group	*P*6_5_22	*P*6_5_22
Unit cell dimensions (Å)	*a* = *b* = 107.13,	*a* = *b* = 107.47,
	*c* = 294.84	*c* = 297.77
Resolution range (Å)	47.14–2.64	47.26–3.30
	(2.79–2.64)	(3.48–3.30)
*R*_sym_ (%)	10.2 (100.5)	17.9 (95.1)
a*R*_*p.i.m*_ (%)	3.8 (38.3)	7.6 (39.8)
b*CC*_*1/2*_	0.998 (0.772)	0.991 (0.684)
Completeness (%)	99.7 (98.1)	99.7 (100.0)
Multiplicity	7.9 (8.2)	5.5 (5.6)
<I/σ(I)>	13.5 (2.1)	10.9 (2.6)
Refined model composition		
Monomers/a. u.	2	2
Protein residues		
Molecule A	M1-K29, T31-L52	S89–R302
	E61-H303	
Molecule B	D5-L52, E61-E300	R87–E300
Water molecules	81	
Thiocyanate ions		2
Glycerol	9	
Wilson *B*-value (Å^2^)	77.5	88.8
Mean B-Value (Å^2^)	93.78	79.03
Model quality indicators		
*R*_work_/*R*_free_ (%)	19.94/22.72	18.82/24.03
Rmsd bond lengths (Å)	0.004	0.004
Rmsd bond angles (°)	0.969	0.863
Estimated coordinate error (Å)	0.24	0.36
^c^Molprobity clash/overall scores	14.84/2.66	8.31/1.98
Ramachandran analysis		
% Favoured	95.4	97.2
% Allowed	4.1	2.8
% Disallowed	0.5	0.0
% rotamer outlier	7.4	3.2
wwPDB ID code	5AE5	5AE4

Numbers in parentheses are for the highest resolution shell.

^a^R_p.i.m_: Precision-indicating merging R factor^59^.

^b^CC_1/2_: Pearson correlation between two half data sets each comprising a random half of the measurements of each unique reflection^60^.

**Table 2:** Melting temperatures (T_m_) and SAXS invariant parameters for *apo-*DntR, H169T-DntR and *apo-*DntR pre-incubated with various concentrations of salicylate.

	**[salicylate]**	**T**_**m**_ (^**o**^**C)**	**R**_**g**_ **(Å)**	**I(0)**	**D**_**max**_ **(Å)**	**Porod volume (nm**^**3**^)
STATIC SAXS						
* apo*-DntR	n.a.	40	38.8 (+/−0.6)	4.97 (+/−0.04)	118	262.8
* *H169T-DntR	n.a	49	41.9 (+/−0.5)		135	
* holo*-DntR	20 μM	n.d.	39.2 (+/−0.4)	5.18 (+/−0.02)	120	262.5
* holo*-DntR	100 μM	51	41.4 (+/−0.4)	5.22 (+/−0.03)	142	262.2
* holo*-DntR	5 mM	n.d.	44.1 (+/−0.4)	5.88 (+/−0.04)	147	296.9
SEC-SAXS						
* apo*-DntR	n.a.	n.d.	39.1 (+/−0.5)	4.90 (+/−0.03)	118	253.3
* holo*-DntR	5 mM	n.d.	41.7 (+/−0.4)	5.20 (+/−0.02)	141	255.2
